# *Lacticaseibacillus rhamnosus* CU262 Attenuates High-Fat Diet–Induced Obesity via Gut–Liver Axis Reprogramming

**DOI:** 10.3390/foods15020332

**Published:** 2026-01-16

**Authors:** Hezixian Guo, Liyi Pan, Linhao Wang, Zongjian Huang, Qiuyi Wu, Jie Wang, Zhenlin Liao

**Affiliations:** College of Food Science, South China Agriculture University, Guangzhou 510642, China

**Keywords:** *L. rhamnosus* CU262, obesity, gut–liver axis, short-chain fatty acids, gut microbiota, hepatic transcriptomics

## Abstract

Obesity is closely linked to dyslipidemia, hepatic injury, and chronic inflammation through disturbances in the gut–liver axis. Here, we evaluated the anti-obesity effects of *L. rhamnosus* (*Lacticaseibacillus rhamnosus*) CU262 in a high-fat diet (HFD) mouse model and elucidated mechanisms using an integrated multi-omics strategy. Male C57BL/6 mice received CU262 during 12 weeks of HFD feeding. Phenotypes, serum/liver biochemistry, gut microbiota (16S rRNA sequencing), fecal short-chain fatty acids (SCFAs), and hepatic transcriptomes (RNA-seq) were assessed. CU262 significantly attenuated weight gain and adiposity; improved serum TC, TG, LDL-C and HDL-C; lowered ALT/AST and FFA; and mitigated oxidative stress and inflammatory imbalance (↓ IL-6/TNF-α, ↑ IL-10). CU262 restored alpha diversity, reduced the *Firmicutes*/*Bacteroidetes* ratio, enriched beneficial taxa (e.g., *Akkermansia*), and increased acetate and butyrate. Liver transcriptomics showed CU262 reversed HFD-induced activation of cholesterol/steroid biosynthesis and endoplasmic reticulum stress, with downregulation of key genes (*Mvk*, *Mvd*, *Fdps*, *Nsdhl*, and *Dhcr7*) and *Pcsk9*, yielding negative enrichment of steroid and terpenoid backbone pathways and enhancement of oxidative phosphorylation and glutathione metabolism. Correlation analyses linked *Akkermansia* and SCFAs with improved lipid/inflammatory indices and repression of cholesterol-synthetic and stress-response genes. These findings demonstrate that CU262 alleviates HFD-induced metabolic derangements via microbiota-SCFA-hepatic gene network reprogramming along the gut–liver axis, supporting its potential as a functional probiotic for obesity management.

## 1. Introduction

Obesity has become a critical global health issue in recent decades, reaching epidemic proportions [1]. The World Health Organization reported that as of 2016, about 39% of adults were overweight, and the prevalence of obesity has nearly tripled since 1975 [1]. Excess adiposity is closely associated with metabolic comorbidities such as hypertension, dyslipidemia, type 2 diabetes, and metabolic-associated fatty liver disease (MAFLD), contributing to a substantial global mortality burden each year [2]. These trends underscore an urgent need for effective interventions and a deeper understanding of obesity’s pathogenesis [3]. In this context, growing evidence highlights the pivotal role of the gut microbiota and the gut–liver axis in metabolic regulation and energy homeostasis [4]. Disruption of the intestinal microbiome and increased gut permeability can lead to heightened translocation of endotoxins into the portal circulation, driving hepatic inflammation and systemic insulin resistance [5]. Notably, a high-fat diet (HFD) is known to induce gut dysbiosis, impair the intestinal barrier, and promote liver fat accumulation and inflammation, thereby creating a deleterious gut–liver feedback loop that exacerbates obesity and metabolic syndrome [6]. This gut–liver crosstalk is now recognized as a key mechanism linking nutritional factors to obesity-related liver and metabolic disorders [7].

Researchers have increasingly turned to probiotics as a promising strategy to counteract obesity and its complications [8]. Probiotic interventions—particularly using lactic acid bacteria such as *Lactobacillus/Lacticaseibacillus* and *Bifidobacterium* species—have shown beneficial effects on host metabolism and adiposity [9]. These commensal microbes can favorably modulate the gut ecosystem and host physiological responses through multiple mechanisms [10]. For example, probiotics can strengthen the intestinal barrier, alter bile acid metabolism, and modulate immune signaling, collectively reducing systemic inflammation and improving metabolic control [11]. In HFD-induced obesity models, supplementation with certain *L. rhamnosus* strains significantly alleviated weight gain and adipose accumulation [12]. Such probiotic treatment has also been shown to attenuate hepatic steatosis and metabolic endotoxemia by suppressing pro-inflammatory pathways (e.g., TLR4) while enhancing fatty-acid oxidation, insulin sensitivity, and gut barrier integrity [13]. Moreover, an important functional outcome of probiotic administration is the enrichment of beneficial gut microbes that produce short-chain fatty acids (SCFAs) [14]. Elevated SCFA levels—such as acetate, propionate, and butyrate—have been linked to improved appetite regulation, reduced inflammation, and enhanced energy metabolism [14]. Indeed, both animal and human studies indicate that *Lactobacillus* probiotics (including *L. rhamnosus*) can increase fecal SCFA concentrations and expand beneficial taxa such as *Bifidobacterium*, which are associated with anti-obesity effects [8]. *L. rhamnosus* CU262 was selected from our isolates for its probiotic potential, including superior acid and bile tolerance and high adhesion capacity [15,16]. Notably, its genome encodes bile salt hydrolases and other metabolic enzymes, suggesting an enhanced capacity to modulate host lipid metabolism compared to other *L. rhamnosus* strains [17].

Despite these advances, there remain important gaps in our understanding of how probiotics exert their anti-obesity effects through the gut–liver axis [18]. Most studies to date have focused on body weight, serum lipids, and gut microbiome profiles but have not systematically interrogated the host’s molecular responses in metabolic organs like the liver [19]. In particular, comprehensive omics approaches that integrate gut microbial community analysis, microbial metabolite (e.g., SCFAs) profiling, and host transcriptomic data are still limited in probiotic research [18]. The absence of such integrated datasets means the mechanistic links between gut microbiota modulation and hepatic gene expression are not fully elucidated [20]. Recent studies suggest that high-throughput transcriptomic analysis is a powerful tool to uncover the signaling pathways and gene networks influenced by probiotic treatment [19]. However, few probiotic studies have employed RNA sequencing alongside microbiome sequencing to decode gut–liver axis interactions [21]. As a result, the precise molecular mechanisms by which specific probiotic strains regulate host metabolism via the gut–liver axis remain incompletely understood [22]. There is a clear need for more systematic, multi-omics investigations to fill this knowledge void and to validate probiotic efficacy from a mechanistic standpoint [18,20].

The present study addresses these gaps by evaluating the effects of *L. rhamnosus* CU262 on HFD-induced obese mice and exploring the underlying gut–liver axis regulation. We established a diet-induced obesity model in mice and administered the CU262 strain to assess its capacity to mitigate weight gain, metabolic derangements, and liver pathology. Importantly, our investigation combines several complementary methodologies: 16S rRNA gene sequencing to characterize shifts in gut microbiota composition, quantification of SCFAs to gauge functional metabolic output of the microbiome, and hepatic transcriptome profiling (RNA-seq) to identify differentially expressed genes and pathways in the liver. By integrating these multi-omics data, we delineate how CU262 modulates the gut microbiome and its metabolites to influence host gene expression and metabolic pathways along the gut–liver axis. To our knowledge, this is one of the first studies to employ a multi-omics approach to unravel a probiotic’s anti-obesity mechanism via the gut–liver axis. Our findings will not only evaluate the efficacy of *L. rhamnosus* CU262 in combating HFD-induced obesity but also illuminate the probiotic’s mode of action through gut–liver signaling networks. This work provides a novel theoretical framework and practical evidence for harnessing specific probiotic strains in the management of obesity and associated metabolic disorders. The insights gained could facilitate the development of targeted microbiome-based interventions and advance our understanding of the gut–liver axis as a therapeutic nexus in metabolic disease.

## 2. Materials and Methods

### 2.1. Bacterial Preparation

A *L. rhamnosus* strain CU262 was isolated from healthy infant feces and identified (GenBank accession PRJNA1244599). The strain was deposited in the Guangdong Microbial Culture Collection Center (GDMCC No. 66018, deposit date 17 March 2025). For preparation of the probiotic inoculum, CU262 was cultivated anaerobically in de Man–Rogosa–Sharpe (MRS) broth at 37 s for 12 h. Cells were harvested by centrifugation (6000× *g*, 5 min, 4 °C) and washed three times with sterile physiological saline. A lyophilized powder was prepared using an optimized freeze-drying protective medium, which contained 8% skim milk, 6% trehalose, 3% fructooligosaccharides, 0.5% sodium L-ascorbate, 1.5% gelatin, and 1% resistant starch (all *w*/*v*). The freeze-drying was conducted for 36 h, yielding a final viable count of ≥1 × 10^9^ CFU/g [23].

### 2.2. Animal Model and Experimental Design

**Figure 1:**
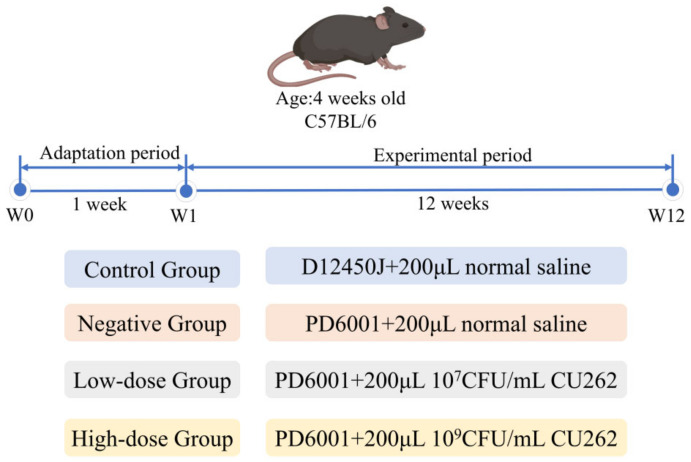
Experimental design for the mouse study.

Twenty-four male C57BL/6J mice (4 weeks old, specific pathogen-free) were obtained from the Southern Medical University Laboratory Animal Center (Guangzhou, China). After one week of acclimatization (housing at 25 ± 2 °C, 12 h light/dark cycle, ad libitum access to standard chow and water), mice were randomly assigned to four groups (*n* = 6 per group) using a computer-generated randomization list and fed for 12 weeks according to the scheme shown in Figure 1, as follows: Control diet (CG)—fed a control low-fat diet (D12450J, 10% kcal from fat) plus daily oral gavage of 200 µL sterile saline; High-fat diet (NG)—fed a high-fat diet (PD6001, 60% kcal from fat) plus 200 µL saline (negative control for obesity); Low-dose CU262 (LD)—fed high-fat diet with a daily gavage of 200 µL CU262 suspension at 1 × 10^7^ CFU/mL; High-dose CU262 (HD)—fed high-fat diet with a daily gavage of 200 µL CU262 at 1 × 10^9^ CFU/mL [24]. The sample size (*n* = 6 per group) was determined based on previous high-fat diet and probiotic intervention studies, which assuming an effect size of approximately difference in body weight gain, provides >80% statistical power at α = 0.05. As shown in Table 1, the semi-purified diets D12450J and PD6001 (Syse bio, China) differ in macronutrient composition: the high-fat diet provided ~60% of energy from fat (34.9 g fat/100 g, 5.24 kcal/g), compared to 10% kcal from fat in the control diet (4.3 g fat/100 g, 3.84 kcal/g), the energy densities (kcal/g) of the diets were derived from these component values. Body weights were recorded weekly during the 12-week feeding period. At the end of the experiment (24 h after the final CU262 or saline gavage), all mice were fasted overnight (12 h) and then euthanized by CO_2_ asphyxiation for sample collection. Blood was collected via orbital sinus or cardiac puncture, allowed to clot, and centrifuged to obtain serum, which was stored at −80 °C until analysis. Major organs (liver, heart, kidneys, spleen) and adipose depots were carefully excised and weighed to calculate organ indices (organ weight as a percentage of body weight). The liver was divided for different analyses: portions were fixed in formalin for histology, immediately frozen in liquid nitrogen for biochemical assays, or immersed in RNA stabilization solution for transcriptomic analysis. In addition, fresh fecal pellets and cecal content were collected aseptically, snap-frozen in liquid nitrogen, and stored at −80 °C for short-chain fatty acid and microbiota analyses. All animal procedures were approved by the Institutional Animal Care and Use Committee, and conducted in accordance with ethical guidelines for animal research.

**Table 1:** Composition differences between diets D12450J and PD6001.

	D12450J		PD6001	
	g/100 g	* kcal%	g/100 g	* kcal%
Protein	76	20	105	20
Carbohydrate	270	70	105	20
Fat	39	10	314	60
kcal/100 g	384		524	

Note: * indicates the percentage of total calories contributed by the corresponding macronutrient in the diet. Energy values were calculated using standard Atwater factors (protein and carbohydrate at 4 kcal/g, fat at 9 kcal/g). Values in parentheses indicate the energy contribution (kcal per 100 g diet) and the percentage of total calories provided by each macronutrient.

### 2.3. Histology

For histological examination, pieces of liver tissue and epididymal white adipose tissue were fixed in 10% neutral buffered formalin, embedded in paraffin, and sectioned at 4 µm. The sections were stained with hematoxylin and eosin (H&E) following standard protocols, and examined under a light microscope (Soptop, Shenzhen, China) for morphological assessment [25]. In liver sections, the degree of steatosis, inflammatory foci, and cellular injury (e.g., ballooning) was qualitatively evaluated. Adipocyte morphology was assessed in epididymal fat sections: adipocyte size (cross-sectional area) was quantified using Image-Pro Plus 6.0 software by measuring the diameters of adipocytes in representative fields and calculating cell area [26]. For each animal, at least five non-overlapping fields were randomly selected, and ≥100 adipocytes per animal were analyzed. An average adipocyte area was obtained for each sample to compare adipocyte hypertrophy among groups. All histological evaluations and image quantifications were performed by an investigator blinded to the group assignments to minimize observational bias.

### 2.4. Serum and Tissue Biochemistry

Blood serum was analyzed for metabolic and inflammatory indicators using commercial assay kits (Nanjing Jiancheng Bioengineering Institute, Nanjing, China, unless otherwise specified) according to the manufacturers’ instructions. Serum lipid profile was evaluated by measuring total cholesterol (TC), triglycerides (TG), low-density lipoprotein cholesterol (LDL-C), and high-density lipoprotein cholesterol (HDL-C). Markers of liver injury, alanine aminotransferase (ALT) and aspartate aminotransferase (AST), were quantified in serum as indicators of hepatocellular damage [27]. Serum free fatty acids (FFA) and total bile acids were also measured. In addition, circulating pro-inflammatory cytokines interleukin-6 (IL-6) and tumor necrosis factor-α (TNF-α), as well as the anti-inflammatory cytokine IL-10, were determined by enzyme-linked immunosorbent assay (ELISA) kits. For liver tissue biochemical assays, approximately 100 mg of fresh liver was homogenized in ice-cold phosphate-buffered saline (PBS) at a 1:9 (*w*:*v*) ratio to produce a 10% liver homogenate. The homogenate was centrifuged at 2500 rpm for 10 min at 4 °C to remove debris, and the supernatant was collected for analysis of hepatic lipids and oxidative stress markers. Hepatic TG and TC levels were measured using colorimetric assay kits, providing an index of lipid accumulation in the liver. Markers of oxidative stress were assessed in the liver homogenate supernatant: superoxide dismutase (SOD) and catalase (CAT) activities were measured (by spectrophotometric rate assays), and malondialdehyde (MDA) content was determined as a marker of lipid peroxidation. All assay results were normalized to protein concentration or tissue weight as appropriate, and expressed as per gram of liver tissue.

The major reagents are illustrated in Table S1.

### 2.5. Short-Chain Fatty Acid Analysis

Short-chain fatty acids (SCFAs) in fecal samples were quantified by gas chromatography–mass spectrometry (GC–MS) [28]. In brief, 100 mg of frozen fecal matter was placed in a 1.5 mL microtube, suspended in 500 µL of deionized water, and spiked with an internal standard (4-methylvaleric acid, final concentration 0.375 mg/mL). The mixture was homogenized vigorously for 1 min with glass beads and then acidified with 100 µL of 15% (*v*/*v*) phosphoric acid to protonate SCFAs. Next, 280 µL of diethyl ether was added as an extraction solvent, and the sample was vortexed and centrifuged (12,000 rpm, 10 min, 4 °C). The organic phase containing SCFAs was carefully collected and used for GC-MS analysis. The analysis was performed on a Thermo Trace 1300 GC system coupled with an ISQ 7000 mass spectrometer (Thermo Fisher Scientific, Waltham, MA, USA). An aliquot (1 µL) of the extract was injected in split mode with a split ratio of 10:1. The injector temperature was set at 250 °C. Chromatographic separation was achieved on an HP-INNOWAX capillary column (30 m × 0.25 mm ID × 0.25 µm film thickness; Agilent, Santa Clara, CA, USA) using high-purity helium as the carrier gas at a constant flow rate of 1.0 mL/min. The GC oven temperature program was as follows: initial temperature 90 °C, then increased to 120 °C at a rate of 10 °C/min, further increased to 150 °C at 5 °C/min, and finally ramped to 250 °C at 25 °C/min and held for 2 min. The transfer line temperature was maintained at 250 °C. The mass spectrometer was operated in electron impact (EI) ionization mode at 70 eV, with the ion source temperature set at 300 °C. Data were acquired in selected ion monitoring (SIM) mode. SCFAs (acetate, propionate, butyrate, iso-butyrate, valerate, isovalerate) were identified by their retention times and characteristic ions, and quantified against calibration curves of authentic standards with correction by the internal standard. Data were expressed as μmol SCFA per gram of wet feces. 

### 2.6. Gut Microbiota Sequencing

Genomic DNA was extracted from cecal content samples using the E.Z.N.A.^®^ Soil DNA Kit (Omega Bio-Tek, Norcross, GA, USA) following the manufacturer’s protocol, which includes efficient lysis of microbial cells from intestinal content. DNA quantity and purity were assessed using a NanoDrop spectrophotometer (Thermo Fisher, Waltham, MA, USA), and integrity was confirmed by 1% agarose gel electrophoresis. The hypervariable regions of the 16S rRNA gene were PCR-amplified from qualified DNA samples using primers 341F (5′-CCTACGGGNGGCWGCAG-3′) and 806R (5′-GACTACHVGGGTATCTAATCC-3′), targeting the V3–V4 region, according to published methods to profile the gut microbial community [29]. After amplification and quality verification, amplicon libraries were prepared by pooling equimolar amounts of PCR products. Library construction included end-repair, A-tailing, and ligation of Illumina sequencing adapters. The prepared libraries were then subjected to high-throughput sequencing on an Illumina NovaSeq 6000 2platform (Illumina Inc., San Diego, CA, USA) with 250 bp paired-end reads, performed by a commercial service provider (Novogene Co., Beijing, China). Raw sequencing reads were demultiplexed and processed using a standard bioinformatics pipeline [30]. Briefly, reads were quality-filtered and merged, and operational taxonomic units or amplicon sequence variants were clustered at 97% sequence identity. Taxonomic classification of sequences was performed by aligning to reference databases for 16S rRNA (SILVA 138.2). Alpha-diversity indices, including Chao1 richness and Shannon diversity, were calculated to evaluate within-sample microbial diversity [31]. Beta-diversity was assessed by principal coordinate analysis (PCoA) based on Bray–Curtis distances to compare global microbiota composition between groups. To identify specific taxa differing in relative abundance between experimental groups, we employed the linear discriminant analysis effect size (LEfSe) algorithm with an LDA score cutoff of 4.0 (*p* < 0.05) for biomarker discovery. This analysis highlights bacterial taxa most likely to explain differences between treatment and control microbiomes. All bioinformatic analyses were carried out using the cloud platform provided by Novogene, and downstream data visualization was performed in R environment.

### 2.7. Liver Transcriptomics

To investigate hepatic gene expression changes, a transcriptomic analysis of liver tissues was performed. Total RNA was extracted from ~50 mg of frozen liver using TRIzol Reagent (Invitrogen, Carlsbad, CA, USA) according to the manufacturer’s instructions. RNA concentration and purity (A_260_/A_280_ ratio) were determined, and RNA integrity was verified by agarose gel electrophoresis to ensure high-quality RNA for sequencing. RNA sequencing (RNA-Seq) was performed using liver samples from all mice in each group (*n* = 6 per group). Library preparation and sequencing were conducted by Novogene Co. (Beijing, China). Briefly, poly(A)^+^ mRNA was enriched from total RNA and reverse-transcribed to cDNA. After end repair, adaptor ligation, and PCR amplification, the cDNA libraries were sequenced on the Illumina NovaSeq 6000 platform, generating paired-end 250 bp reads. Raw sequence data were filtered to remove low-quality reads and adapters, and then aligned to the GRCm39 mouse reference genome using HISAT2 v2.2.1 [32]. Gene expression levels were quantified (e.g., in fragments per kilobase of transcript per million mapped reads, FPKM). Differential expression analysis between groups (HFD control vs. normal diet control, and probiotic-treated vs. HFD control) was carried out using the DESeq2 package (v1.38) within the R statistical environment (v4.3.0) [33]. Genes with a fold change > 2 (i.e., >2 or <0.5 in expression ratio) and a *p* value < 0.05 (after false discovery rate correction) were considered significantly differentially expressed. To interpret the biological significance of the expression changes, identified differentially expressed genes (DEGs) were subjected to functional enrichment analysis. Gene Ontology (GO) enrichment (biological process, molecular function, cellular component) and Kyoto Encyclopedia of Genes and Genomes (KEGG) pathway analysis were performed to find overrepresented functional categories among the DEGs [34]. Additionally, Gene Set Enrichment Analysis (GSEA) was applied using the whole transcriptome expression data to detect coordinated changes in predefined gene sets/pathways associated with metabolism, inflammation, and stress responses [35]. These analyses helped to pinpoint the key metabolic and regulatory pathways modulated by the HFD and by the CU262 intervention in the liver.

### 2.8. Statistical Analysis

The sample size (*n* = 6 per group) was determined based on previous high-fat diet and probiotic intervention studies, which, assuming an effect size in body weight gain, provides >80% statistical power at α = 0.05. For parametric analyses, data were verified to approximate normal distribution (Shapiro–Wilk test) and equal variances (Levene’s test). All data are expressed as the mean ± standard deviation (SD). GraphPad Prism 9.5 (USA) was used for statistical analysis and graphing. For comparisons involving more than two groups, one-way analysis of variance (ANOVA) was performed, followed by Tukey’s post hoc test to determine significance between groups. For high-dimensional analyses, *p* values were adjusted for multiple comparisons using the Benjamini–Hochberg false discovery rate (FDR) procedure. For all tests, a *p* value < 0.05 was considered statistically significant.

## 3. Results

### 3.1. CU262 Mitigates HFD-Induced Obesity and Adipose Tissue Hypertrophy

**Figure 2:**
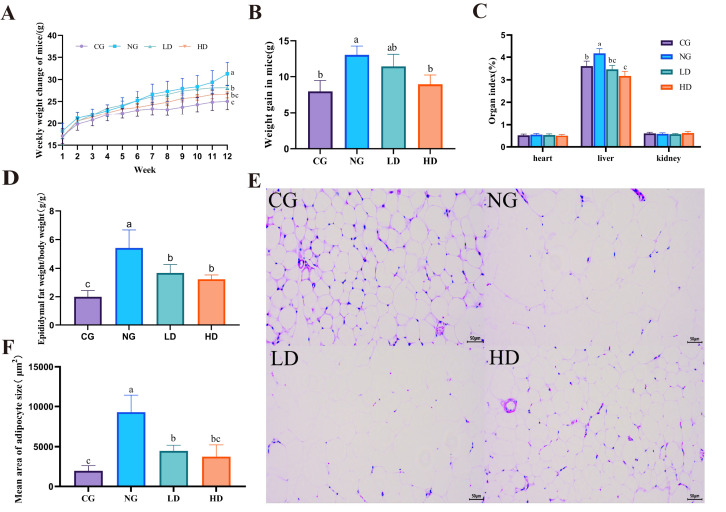
Effects of *L. rhamnosus* CU262 on body weight, organs, and adipose tissue in HFD mice. (**A**) Body-weight trajectory over the 12-week experimental period; (**B**) Body-weight gain before and after the experiment; (**C**) Organ indices (heart, liver, kidney) after 12 weeks; (**D**) Epididymal fat mass after 12 weeks; (**E**) Representative H&E-stained sections of epididymal adipose tissue after 12 weeks (scale bar, 50 μm); (**F**) Mean adipocyte area in epididymal fat after 12 weeks. (Different lowercase letters indicate significant differences among groups (*n* = 6, *p* < 0.05); CG, control group; NG, negative control group; LD, low-dose CU262 group; HD, high-dose CU262 group).

*Lacticaseibacillus rhamnosus* CU262 supplementation significantly protected against HFD-induced obesity in mice. By the end of 12 weeks, HFD-fed mice (NG) gained markedly more weight than the control diet group (CG), whereas CU262-treated mice showed a much smaller increase (Figure 2A,B). In particular, the high-dose CU262 group (HD) gained ~30% less body weight than the HFD-only group (*p* < 0.05), indicating an impressive attenuation of diet-induced weight gain (Figure 2B). Consistent with this blunted weight gain, CU262 also reduced fat accumulation. HFD caused an expansion of adipose depots and enlarged livers (reflected by higher epididymal fat mass and liver weight index in NG vs. CG), but CU262 treatment significantly lowered these indices toward normal levels (Figure 2C,D).

Histological evidence further confirmed the anti-obesity effect of CU262. H&E-stained epididymal adipose sections from HFD-only mice revealed adipocyte hypertrophy—very large, lipid-laden fat cells with fewer cells per field—characteristic of excessive fat storage (Figure 2E). In contrast, CU262-treated mice (especially the HD group) had markedly smaller adipocytes and more cells per area, indicating reduced fat cell hypertrophy (Figure 2E,F). Quantitatively, the average adipocyte cross-sectional area in the HD group was significantly lower than in HFD controls (*p* < 0.05, Figure 2F). These results demonstrate that *L. rhamnosus* CU262 effectively alleviates HFD-induced obesity, as evidenced by reduced weight gain and diminished adipose tissue expansion. This finding is in line with recent reports that certain *L. rhamnosus* strains can improve obesity-related phenotypes in HFD-fed mice (e.g., reducing weight gain and adiposity) [36]. Probiotics are thought to exert anti-obesity effects by inhibiting adipogenesis and lipid storage in adipose tissue, which likely contributes to the smaller adipocyte size and lower fat mass observed in CU262- treated mice [37].

### 3.2. CU262 Improves Serum Lipid Profile, Liver Function and Inflammation

**Figure 3:**
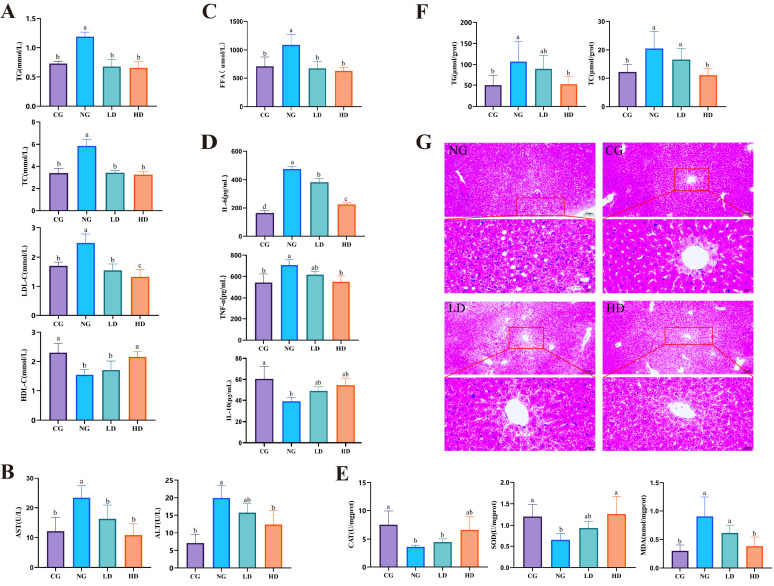
Effects of *L. rhamnosus* CU262 on serum parameters and the liver in HFD mice. (**A**) Serum lipid profile after 12 weeks (from top to bottom: total cholesterol, triglycerides, low-density lipoprotein cholesterol, high-density lipoprotein cholesterol); (**B**) Serum aspartate transaminase (AST) and alanine aminotransferase (ALT) after 12 weeks; (**C**) Serum free fatty acids (FFA) after 12 weeks; (**D**) Serum inflammatory cytokines after 12 weeks (from top to bottom: IL-6, TNF-α, and IL-10); (**E**) Total cholesterol and triglycerides in liver homogenates after 12 weeks; (**F**) Antioxidant enzyme activities (superoxide dismutase, SOD; catalase, CAT) and malondialdehyde (MDA) in liver homogenates after 12 weeks; (**G**) Representative H&E-stained liver sections after 12 weeks (scale bar, 100 μm). (Different lowercase letters indicate significant differences among groups (*n* = 6, *p* < 0.05); CG, control group; NG, negative control group; LD, low-dose CU262 group; HD, high-dose CU262 group).

Chronic high-fat feeding led to pronounced dyslipidemia, liver injury, and systemic inflammation in the mice—all of which were significantly ameliorated by CU262 supplementation. As shown in Figure 3A, the HFD caused a significant increase in serum total cholesterol (TC), triglycerides (TG), and LDL-C, along with a decrease in HDL-C (NG vs. CG, *p* < 0.05). These changes reflect the typical lipid disturbances of diet-induced obesity. Remarkably, CU262 intervention restored a healthier serum lipid profile. Both low- and high-dose CU262 groups had lower TC, TG, and LDL-C than HFD-only mice, with the high-dose achieving significant reductions (*p* < 0.05, LD, HD vs. NG). HDL-C levels were also higher in CU262-treated mice (especially HD), nearly normalizing the lipid profile (Figure 3A). This indicates that CU262 helps counteract HFD-induced hyperlipidemia. Consistent with our findings, clinical studies have reported that probiotic supplementation can significantly improve blood lipids, reducing cholesterol, TG and LDL levels in metabolic disorder patients [38].

High-fat diet also caused liver damage and metabolic stress, evidenced by elevated serum liver enzymes and free fatty acids, as well as an imbalance in inflammatory cytokines. HFD-fed mice showed significantly higher alanine aminotransferase (ALT) and aspartate aminotransferase (AST) levels compared to controls, indicating hepatic injury (Figure 3B). CU262 supplementation provided strong hepatoprotection: ALT and AST in CU262-treated groups (especially HD) were dramatically lower than in HFD-only mice, dropping to near the values of lean controls (Figure 3B). This suggests that CU262 largely prevented HFD-induced liver damage, in line with reports that probiotics can reduce liver enzymes in metabolic fatty liver disease [39]. The HFD control (NG) group also exhibited a marked increase in circulating free fatty acids (FFA), which can promote lipotoxicity and insulin resistance, whereas CU262-treated mice had significantly lower FFA levels (Figure 3C). Moreover, HFD induced a proinflammatory cytokine profile—elevated IL-6 and TNF-α and suppressed IL-10—consistent with chronic low-grade inflammation. CU262 reversed this imbalance, as IL-6 and TNF-α were decreased and the anti-inflammatory IL-10 was increased in treated mice (Figure 3D). High-dose CU262 in particular brought IL-6/TNF-α down and IL-10 up relative to HFD alone (*p* < 0.05), indicating an attenuation of obesity-associated systemic inflammation. These immunomodulatory effects of CU262 are consistent with the known ability of probiotics to reduce systemic inflammation in vivo [40].

In the liver, CU262 supplementation alleviated HFD-induced steatosis and oxidative stress [41]. HFD-only mice had significantly higher hepatic triglyceride and cholesterol contents than controls (indicating fatty liver) [40], and they showed impaired antioxidant defenses—liver homogenates from HFD mice had much lower superoxide dismutase (SOD) and catalase (CAT) activities and elevated malondialdehyde (MDA, a lipid peroxidation marker) [42] (Figure 3E,F). CU262 treatment markedly reversed these pathological changes. High-dose CU262 mice had much lower hepatic TG and cholesterol levels (nearly restored to normal) [43], along with significantly increased SOD/CAT activities and reduced MDA compared to HFD-alone (Figure 3E,F, *p* < 0.05 HD vs. NG). This indicates that CU262 protected the liver from fat accumulation and oxidative damage, likely by reducing the influx of FFA and inflammatory signals to the liver.

Histopathological examination further corroborated these findings (Figure 3G). Liver sections from HFD-fed mice showed severe macrovesicular steatosis (large fat vacuoles in hepatocytes), inflammatory cell infiltration, and hepatocellular ballooning degeneration—features of steatohepatitis. In contrast, livers from CU262-treated mice (especially the HD group) had far fewer fat vacuoles and only mild, scattered inflammation, with preservation of normal liver architecture. This visual evidence confirms that CU262 dramatically attenuated HFD-induced liver injury, paralleling the improvements in biochemical markers. Altogether, these results demonstrate that *L. rhamnosus* CU262 effectively improves the serum lipid profile, protects liver function, and reduces systemic inflammation in HFD-fed mice [39]. Moreover, the probiotic’s multi-faceted benefits corroborate the evidence that targeting the gut–liver axis via probiotics is an effective strategy to combat diet-induced metabolic disorders [44].

### 3.3. CU262 Elevates Short-Chain Fatty Acids and Reshapes Gut Microbiota Composition

**Figure 4:**
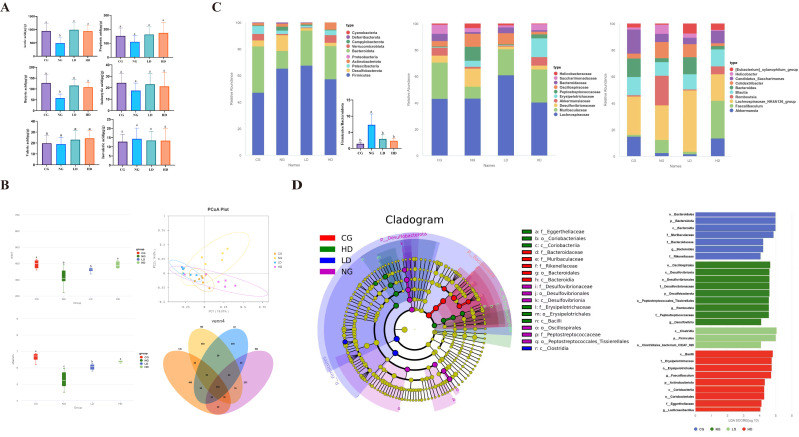
Effects of *L. rhamnosus* CU262 on fecal short-chain fatty acids and gut microbiota in HFD mice. (**A**) Fecal short-chain fatty acid concentrations after 12 weeks (from left to right, top to bottom: acetate, propionate, butyrate, isobutyrate, valerate, and isovalerate); (**B**) Gut microbial alpha diversity (Chao1 richness, top left; Shannon diversity, bottom left) and beta diversity (PCoA based on Bray–Curtis distances, top right) after 12 weeks, and Venn diagram of ASVs among groups (bottom right); (**C**) Relative abundances of gut microbes at different taxonomic ranks after 12 weeks (from left to right: phylum, family, and genus), and *Firmicutes*/*Bacteroidetes* ratio at the phylum level; (**D**) LEfSe-based cladogram and LDA score distribution of differential taxa after 12 weeks (|LDA score| = 4). (Different lowercase letters indicate significant differences among groups (*n* = 6, *p* < 0.05); CG, control group; NG, negative control group; LD, low-dose CU262 group; HD, high-dose CU262 group).

To investigate gut–liver axis mechanisms, we analyzed fecal short-chain fatty acids (SCFAs) and gut microbiota in each group. High-fat feeding caused a significant reduction in beneficial SCFAs: HFD mice had much lower fecal acetate and butyrate concentrations than controls (*p* < 0.05, NG vs. CG), along with trends of decreased propionate and other minor SCFAs (Figure 4A). Importantly, CU262 supplementation increased SCFA production. High-dose CU262 mice showed robust elevations in acetate and butyrate levels compared to HFD-only (Figure 4A, *p* < 0.05), essentially reversing the SCFA deficit caused by HFD. Butyrate in the HD group, for example, was restored to a level comparable to lean controls. The low-dose CU262 group also showed an upward trend in SCFAs, though changes were most pronounced in HD. This restoration of SCFAs is beneficial, as these metabolites serve as energy substrates and signaling molecules that improve host metabolism. In particular, SCFAs like acetate and butyrate can promote fatty acid oxidation and strengthen gut barrier integrity [45]. Butyrate also activates hepatic AMPK, shifting metabolism away from fat storage toward fat burning [46]. Thus, the increased availability of SCFAs in CU262-treated mice likely contributed to their improved metabolic outcomes [47].

We next examined the gut microbiota diversity and community structure. Obesity and HFD are often associated with reduced microbial diversity [48], and our results reflected this. HFD-fed mice (NG) had significantly lower alpha-diversity compared to controls—reduced species richness (Chao1 index) and Shannon diversity (*p* < 0.05, NG vs. CG; Figure 4B)—indicating a loss of gut microbial richness due to the high-fat diet. Notably, CU262 halted and reversed this trend. Mice given CU262, especially the high dose, showed higher Chao1 and Shannon indices than untreated HFD mice (Figure 4B), approaching the diversity levels of the control group. In fact, alpha-diversity in the HD group was not significantly different from the control diet group, suggesting that CU262 preserved a more eubiotic microbiota despite HFD challenge. Similarly, beta-diversity analysis (PCoA of Bray–Curtis distances) revealed distinct clustering of microbial communities: the HFD-alone group’s microbiota diverged substantially from that of controls, whereas CU262-treated groups shifted closer to the control cluster (Figure 4B). This indicates that CU262 modulated the overall microbiome structure toward a healthier composition, consistent with probiotics partially restoring a dysbiotic microbiota [49]. A Venn diagram of amplicon sequence variants (ASVs) further showed that CU262 mice shared more microbial species with controls than HFD-alone did (Figure 4B), underscoring the probiotic’s role in preserving microbial richness and stability.

At the phylum level, our 16S rRNA sequencing data showed that HFD feeding led to the characteristic shift commonly reported in obesity: an increased ratio of *Firmicutes* to *Bacteroidetes* (F/B ratio) in the gut microbiota of NG mice relative to controls. Specifically, the relative abundance of *Firmicutes* rose while *Bacteroidetes* declined, resulting in a significantly elevated F/B ratio in HFD mice (Figure 4C). A high F/B ratio is a well-recognized marker of gut dysbiosis in obesity [50]. Treatment with CU262 significantly lowered the *Firmicutes*/*Bacteroidetes* ratio in HFD-fed mice (Figure 4C). In the HD group, the F/B ratio was reduced toward the lean control level, indicating a corrective effect on the dysbiosis induced by the high-fat diet. This shift likely reflects both a decrease in certain *Firmicutes* and an enrichment of *Bacteroidetes* or other beneficial phyla due to CU262.

At the community level, HFD skewed the gut microbiota toward a dysbiotic profile. The characteristic *Firmicutes*-to-*Bacteroidetes* (F/B) ratio—a hallmark of obesity-associated dysbiosis—was significantly elevated in HFD-fed mice relative to controls (Figure 4C). Treatment with CU262 effectively lowered the F/B ratio in HFD-fed mice, bringing it closer to the lean control level (Figure 4C). This suggests a correction of the HFD-induced imbalance between major phyla. HFD also increased the relative abundance of potentially harmful bacteria while depleting beneficial ones. For example, HFD mice showed overgrowth of opportunistic taxa such as *Romboutsia* (family *Peptostreptococcaceae*) and *Helicobacter* (family *Helicobacteraceae*), and a reduction in beneficial groups like *Muribaculaceae* and *Akkermansia*. Such dysbiotic shifts (enrichment of pro-inflammatory microbes and loss of beneficial commensals) are known to impair the gut barrier and promote inflammation [51,52]. CU262 supplementation reversed many of these changes. In CU262-treated mice, the harmful HFD-enriched taxa were suppressed, and beneficial bacteria flourished. The F/B ratio dropped toward normal, and total microbial composition shifted to resemble that of healthy controls (Figure 4B,C). Notably, the genus *Akkermansia* was significantly enriched in both low- and high-dose CU262 groups compared to HFD-alone, recovering to nearly control levels.

*Akkermansia*’s resurgence is particularly noteworthy given this bacterium’s known association with metabolic health. *Akkermansia* muciniphila is a mucin-degrading microbe that has been linked to improved insulin sensitivity and reduced adiposity in both mice and humans [53]. It also produces SCFAs (especially acetate) and strengthens gut barrier integrity, actions that can reduce systemic inflammation and metabolic endotoxemia [54]. The significant increase in *Akkermansia* (along with other SCFA-producers) in CU262-treated mice likely underpins the higher butyrate levels and anti-inflammatory milieu observed in those groups. In addition to *Akkermansia*, CU262 significantly enriched other beneficial SCFA-producing genera such as *Faecalibaculum* and *Lactobacillus*, which would contribute to butyrate production and gut health. Meanwhile, pro-inflammatory and potentially pathogenic genera that were promoted by HFD—including *Romboutsia*, *Colidextribacter*, and *Desulfovibrio*—were diminished in CU262-treated mice. Taken together, these data demonstrate that CU262 reshaped the gut microbiota toward a healthier profile: microbial diversity was increased, beneficial commensals (notably fiber-fermenting, SCFA-producing bacteria) were promoted, and deleterious bacteria associated with endotoxin release and inflammation were suppressed. Such microbiota remodeling is strongly associated with protection against obesity and inflammation [53].

### 3.4. CU262 Reprograms Hepatic Gene Expression Profiles

**Figure 5:**
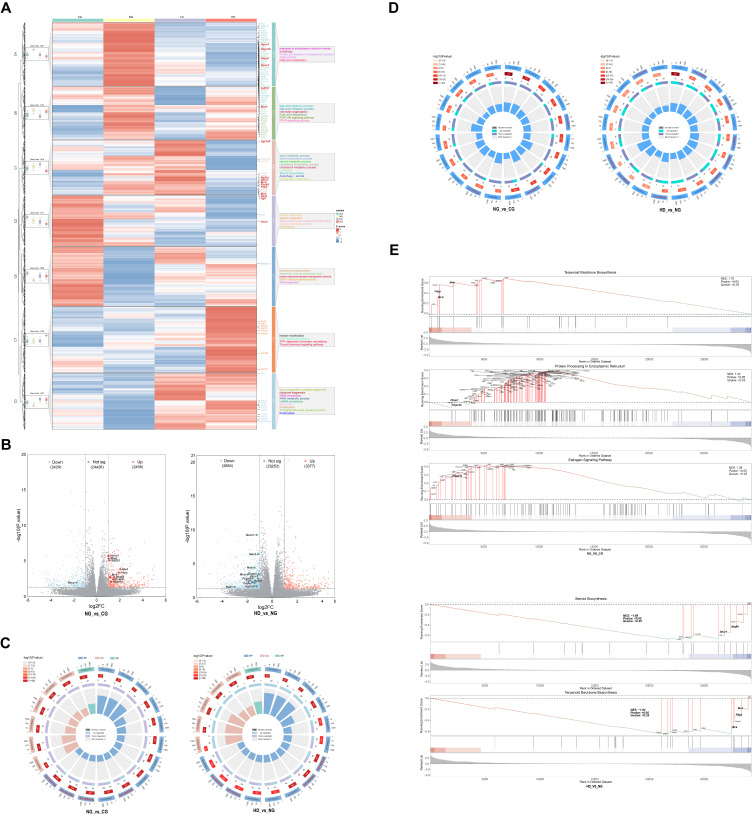
Transcriptomic analysis of hepatic gene expression in HFD-fed mice treated with *L. rhamnosus* CU262. (**A**) Heatmap of liver gene expression (FPKM > 1). Clusters 1–7 were obtained by mfuzz clustering of transcriptomic data; box plots indicate expression trends of each cluster across groups. The right panel lists obesity-related GO/KEGG pathways and key genes in each cluster; genes in bold red are significantly different in NG vs. CG and HD vs. NG (*p* < 0.05, |log2FoldChange| ≥ 1). (**B**) Volcano plots of hepatic differentially expressed genes; labeled genes are key genes in obesity-related pathways. (**C**) GO enrichment circle plot of hepatic DEGs; red labels mark pathways containing obesity-related significant DEGs. (**D**) KEGG enrichment circle plot of hepatic DEGs; red labels mark pathways containing obesity-related significant DEGs. (**E**) GSEA enrichment plots of hepatic DEGs; bolded gene names denote genes highly related to obesity (|NES| > 1, nominal *p* < 0.05, FDR q < 0.25). (CG, control group; NG, negative control group; LD, low-dose CU262 group; HD, high-dose CU262 group; RNA-Seq was conducted with *n* = 6 biological replicates per group; the specific statistics are applied in Table S2).

To elucidate how CU262 influences host metabolism via the gut–liver axis, we performed transcriptomic analysis of liver tissues. High-throughput RNA-seq revealed that HFD consumption induced broad changes in hepatic gene expression, many of which were counter-regulated by CU262 treatment [55]. Unsupervised clustering of liver transcriptomes (Figure 5A) showed that HFD-only mice exhibited coordinated upregulation of a cluster of genes involved in cholesterol biosynthesis (mevalonate pathway) and endoplasmic reticulum (ER) stress responses, compared to normal diet controls [56]. In mice receiving CU262 (especially high-dose), these HFD-induced transcriptional abnormalities were markedly reversed (Figure 5A). Functional annotation indicated that CU262 restored the expression of genes in key metabolic pathways typically disrupted in obesity—including fatty acid *β*-oxidation, *PPAR* signaling, oxidative phosphorylation, and glutathione metabolism [57]. In particular, several critical regulators that were aberrantly expressed under HFD were brought back toward normal levels by CU262. For instance, HFD-fed mice had sharply elevated hepatic expression of cholesterol-synthesis enzymes (*Mvk*, *Mvd*, *Fdps*, *Nsdhl*), which would drive excess cholesterol production and contribute to fatty liver. Strikingly, in CU262-treated mice these same genes were significantly downregulated relative to HFD-alone, often to levels even lower than controls (Figure 5A). This suppression of cholesterol biosynthetic genes suggests reduced de novo cholesterol synthesis in the liver, which is beneficial for preventing steatosis and hypercholesterolemia. Consistent with this, we observed that *Pcsk9*—a key regulator of cholesterol homeostasis—was strongly downregulated by CU262 (HD vs. NG). HFD had induced *Pcsk9* (NG vs. CG), which promotes LDL receptor degradation and elevates blood LDL-C [58]; CU262 largely prevented this increase, implying improved LDL receptor availability and cholesterol clearance in the liver. In addition to lipid metabolism genes, CU262 also normalized stress-response genes. HFD upregulated ER chaperones (e.g., *Hspa1b*, *Hyou1*, and *Manf*), indicating activation of the unfolded protein response due to metabolic stress. High-dose CU262 significantly reduced the expression of these ER stress markers (versus HFD-alone), suggesting an alleviation of HFD-induced ER stress. Collectively, these gene-expression changes demonstrate that CU262 “reprogrammed” the hepatic transcriptome, counteracting HFD’s effects by turning off genes in cholesterol/fat synthesis pathways and stress responses, while likely enhancing genes for fatty acid oxidation and antioxidant defense. This coordinated normalization at the gene level provides a mechanistic basis for the improved liver phenotypes observed with CU262 treatment.

It is notable that microbiota-derived SCFAs might contribute to some of these hepatic gene effects. SCFAs (such as butyrate) are known to activate *AMPK* and thereby inhibit hepatic cholesterol and fat synthesis [46]. In our study, the substantial increase in butyrate in CU262-treated mice coincided with strong suppression of the mevalonate–cholesterol synthesis genes, suggesting that SCFA signaling could be one factor mediating the probiotic’s impact on liver gene expression.

Differential expression and pathway analyses reinforced these findings (Figure 5B–E). In the HFD vs. control comparison (NG vs. CG), numerous genes involved in cholesterol biosynthesis (e.g., *Mvd*, *Fdps*, *Mvk*, *Nsdhl*) were among the most significantly upregulated by HFD (log_2_ fold-change > 1, *p* < 0.05), along with *Pcsk9* and stress-related genes like *Hspa1b* and *Hyou1* (Figure 5B). These changes highlight an overactive cholesterol synthesis pathway and elevated stress response in obese livers. Conversely, in the probiotic-treated vs. HFD comparison (HD vs. NG), those same genes were among the most significantly downregulated in CU262-treated livers (Figure 5B). For example, *Mvk* and *Fdps* expression was dramatically lower in HD than NG, confirming that CU262 directly dampened the HFD-driven cholesterogenic program. *Pcsk9* was also significantly downregulated, which would preserve hepatic LDL receptors and promote cholesterol removal from the bloodstream. Likewise, stress-inducible genes (*Hspa1b*, *Manf*, etc.) were down, indicating relief of HFD-induced ER stress. These targeted gene changes show that CU262 intervention hit critical nodes of metabolic dysregulation, suppressing the HFD-induced sterol synthesis and unfolded protein response. As a result, hepatic cholesterol accumulation and inflammatory stress signaling would be expected to decrease, consistent with the improved liver lipid and enzyme levels we observed.

Gene enrichment analyses further corroborated the protective effects of CU262 on liver metabolism. Gene Ontology (GO) analysis showed that HFD livers were significantly enriched in processes related to sterol biosynthesis, fatty acid metabolic process, and oxidation–reduction (reflecting lipid overload and oxidative stress). In contrast, high-dose CU262 treatment yielded the opposite pattern: genes in sterol/cholesterol biosynthetic processes were significantly depleted (downregulated), while genes in pathways like fatty acid *β*-oxidation, oxidative phosphorylation, and glutathione metabolism were enriched (upregulated) in CU262 vs. HFD (Figure 5C). In other words, CU262 reversed the HFD-induced transcriptional shift—turning off pathways of cholesterol/fat synthesis and turning on pathways of fatty acid utilization and antioxidant defense. Similarly, KEGG pathway analysis revealed that HFD predominantly upregulated metabolic pathways for cholesterol/steroid biosynthesis and glycolysis (consistent with lipogenesis and energy surplus), whereas CU262 suppressed these aberrant pathways and significantly enhanced pathways for oxidative phosphorylation, glutathione metabolism, and other mitochondrial/Detoxification functions (Figure 5D). These KEGG results indicate that CU262 shifted the liver’s metabolic program from a lipid-producing, pro-oxidant state toward a more oxidative, energy-burning and antioxidative state.

Gene Set Enrichment Analysis (GSEA) provided a global view of how entire pathways were coordinately affected. In NG vs. CG (HFD vs. control), GSEA showed a strong positive enrichment of the terpenoid backbone biosynthesis pathway (upstream mevalonate/cholesterol synthesis), confirming that HFD activated the cholesterogenic axis (Figure 5E). Conversely, in HD vs. NG (probiotic vs. HFD), cholesterol/sterol biosynthesis pathways were among the most negatively enriched gene sets—i.e., significantly downregulated by CU262 (Figure 5E). Key enzymes in these pathways (*Mvk, Fdps, Nsdhl, Dhcr7*, etc.) had much lower expression in CU262-treated mice than in HFD mice, reinforcing that CU262 broadly repressed the cholesterol synthesis program. On the other hand, oxidative metabolism pathways (such as oxidative phosphorylation) and glutathione metabolism were positively enriched in CU262 vs. HFD, whereas they were negatively enriched in HFD vs. control. This “mirror-image” pattern demonstrates a striking pathway-level reversal: CU262 turned off the lipogenic and stress pathways induced by HFD, and turned on pathways for lipid oxidation, mitochondrial function, and antioxidant capacity.

Overall, the hepatic transcriptomic data provide a mechanistic underpinning for CU262’s metabolic benefits. By downregulating key enzymes in cholesterol/fat synthesis (and *Pcsk9*) and upregulating pathways for fatty acid oxidation and antioxidative defense, CU262 reduced lipid accumulation and stress in the liver, explaining the improvements in serum and hepatic markers. These findings are consistent with emerging multi-omics studies showing that probiotics (or their metabolites) can beneficially remodel host hepatic gene expression profiles [56]. In our case, the combined influence of a restored gut microbiota (e.g., enriched Akkermansia) and its metabolites (e.g., elevated SCFAs) appears to drive the observed gene-expression reprogramming, illustrating how modulation of the gut–liver axis at the molecular level translates into metabolic improvements.

### 3.5. Integrative Correlation Analysis Reveals Gut–Liver Axis Interactions

**Figure 6:**
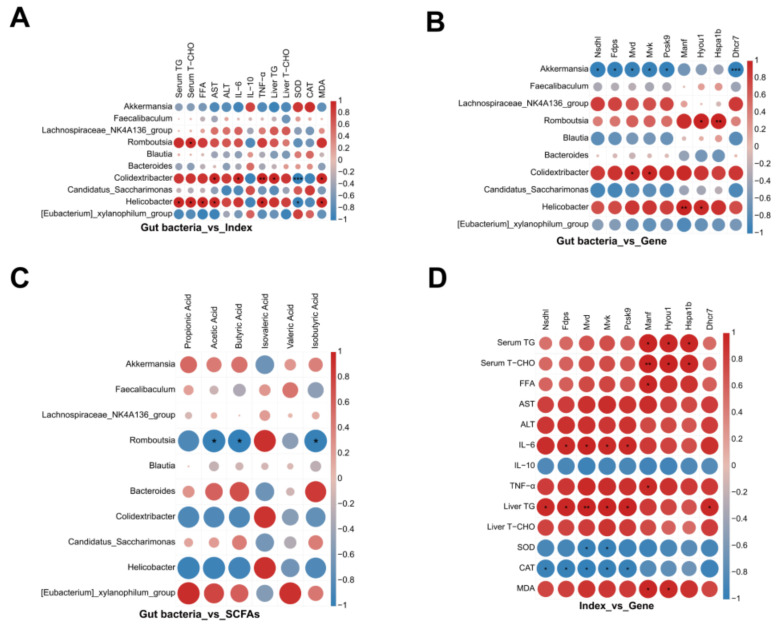
Correlations among gut microbiota, serum and hepatic indices, fecal short-chain fatty acids, and key hepatic differentially expressed genes in HFD-fed mice treated with *L. rhamnosus* CU262. (**A**) Correlations between gut genera and serum/liver indices; (**B**) Correlations between gut genera and key hepatic differentially expressed genes; (**C**) Correlations between gut genera and fecal short-chain fatty acids; (**D**) Correlations between serum/liver indices and key hepatic differentially expressed genes. (* *p* < 0.05, ** *p* < 0.01, *** *p* < 0.001; the specific statistics are applied in Table S3).

Finally, to integrate our multi-omics data, we performed a comprehensive correlation analysis linking gut microbiota abundances, fecal SCFA levels, hepatic gene expression, and host metabolic indices across all animals. The resulting correlation heatmaps (Figure 6A–D) reveal several important gut–liver axis relationships.

Gut microbes vs. host metabolic indices (Figure 6A): The abundance of *Akkermansia* exhibited strong negative correlations with multiple obesity-related indicators. Mice with higher *Akkermansia* levels tended to have lower serum TG and TC, lower FFA, and lower hepatic lipid content, as well as higher anti-inflammatory and antioxidant measures (e.g., increased serum IL-10 and hepatic SOD/CAT activities). Conversely, genera that were enriched by HFD—including *Romboutsia*, *Colidextribacter*, and *Helicobacter*—showed the opposite pattern. High levels of these bacteria were associated with higher serum lipids and inflammatory cytokines (IL-6, TNF-α), and lower IL-10 and antioxidant enzyme levels. These results affirm that beneficial bacteria like *Akkermansia* correlate with a healthier metabolic profile (less hyperlipidemia and inflammation) [59], whereas the presence of a dysbiotic HFD-associated microbiota correlates with worse metabolic outcomes [5,51,60,61].

Gut microbes vs. liver gene expression (Figure 6B): Microbiota composition was also linked to hepatic gene expression patterns. Notably, *Akkermansia* abundance was strongly inversely correlated with the expression of the cholesterol synthesis genes *Nsdhl*, *Fdps*, *Mvd*, and *Mvk*, as well as *Pcsk9* (Spearman r < 0, *p* < 0.05). In other words, mice harboring more *Akkermansia* tended to have lower hepatic expression of these lipogenic and cholesterol-regulating genes. In contrast, the HFD-favored genera (*Romboutsia*, *Colidextribacter*, *Helicobacter*) showed significant positive correlations with those same hepatic genes—higher abundance of these bacteria associated with higher expression of *Nsdhl*, *Fdps*, *Mvk*, etc.—and they also positively correlated with ER stress genes like *Hyou1* and *Hspa1b*. These opposing trends suggest a tug-of-war between beneficial vs. harmful microbiota signals: an *Akkermansia*-rich gut may send signals that restrain hepatic cholesterol synthesis (for example, via SCFA-activated *AMPK* pathways [62]), whereas a dysbiotic gut microbiota may exacerbate liver lipogenesis and stress gene expression.

Gut microbes vs. SCFA levels (Figure 6C): The correlation analysis confirmed that SCFA production is closely tied to microbiota composition. *Akkermansia* and certain fiber-degrading commensals were positively correlated with fecal SCFA levels—mice with more *Akkermansia* (and, e.g., *the [Eubacterium]xylanophilum group*) had higher acetate, propionate, and butyrate concentrations (*p* < 0.05). On the other hand, *Romboutsia*, *Colidextribacter*, and *Helicobacter* abundances correlated negatively with these SCFAs (r < 0, *p* < 0.05), meaning animals dominated by those bacteria had lower output of beneficial SCFAs (Figure 6C). In essence, a probiotic-enhanced microbiota was associated with more SCFA production, while an HFD-induced dysbiotic microbiota produced less. This is consistent with the known loss of SCFA-producing bacteria under high-fat dysbiosis and the restoration of SCFA levels by probiotic interventions [63,64].

Host metabolic markers vs. liver genes (Figure 6D): We also observed strong links between hepatic gene expression and systemic metabolic markers. The hepatic mRNA levels of cholesterol synthesis genes (*Mvk*, *Fdps*, *Nsdhl*, etc.) were positively correlated with serum and liver lipid levels—livers that expressed more of these lipogenic genes tended to belong to mice with higher serum TG, higher cholesterol, and greater hepatic lipid accumulation. Similarly, the expression of ER stress genes (*Hspa1b*, *Hyou1*, etc.) correlated positively with proinflammatory cytokines IL-6 and TNF-α. In contrast, anti-inflammatory and antioxidant markers were negatively correlated with the lipogenic and stress gene expression: for instance, mice with higher IL-10 or higher hepatic SOD/CAT activity tended to have lower expression of *Mvk*, *Fdps*, and other cholesterol synthesis/ER stress genes. These relationships indicate that activation of hepatic cholesterol synthesis and ER stress pathways is strongly associated with worse metabolic and inflammatory outcomes (hyperlipidemia and systemic inflammation) [65,66]. Conversely, lowering the expression of those pathways—as achieved by CU262—is linked to improved metabolic health (lower lipids and inflammation, higher IL-10 and antioxidant status).

**Figure 7:**
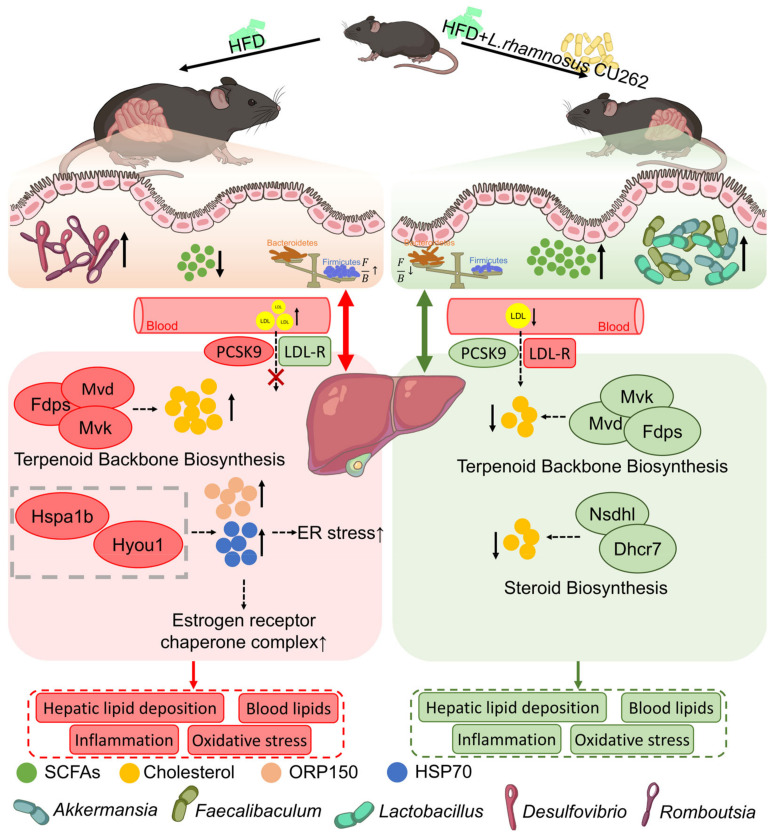
Proposed mechanism by which *L. rhamnosus* CU262 alleviates HFD-induced obesity via modulation of the gut–liver axis. Red ovals/rectangles indicate upregulated genes/indices; green ovals/rectangles indicate downregulated genes/indices. Black arrows: ↑ upregulation; ↓ downregulation. Dashed arrows represent reaction or metabolic processes.

In summary, this integrative correlation analysis paints a coherent picture of gut–liver crosstalk in our model. The proliferation of beneficial gut microbes (like *Akkermansia*), promoted by CU262, was associated with higher SCFA production, reduced expression of hepatic cholesterol synthesis and stress genes, and improvements in systemic lipid profiles and inflammation. On the other hand, the presence of a dysbiotic microbiota (rich in *Romboutsia*, *Helicobacter*, etc., as in untreated HFD mice) was linked to lower SCFAs, upregulated liver lipogenic/stress pathways, and worse metabolic and inflammatory markers. These findings align with emerging evidence that the gut microbial ecosystem and its metabolites are tightly coupled to host metabolic homeostasis [67]. In particular, the enrichment of A. muciniphila and other SCFA-producing bacteria by CU262 appears central to its anti-obesity mechanism, echoing studies where A. muciniphila or butyrate supplementation protected against HFD-induced obesity by strengthening the gut barrier and enhancing fatty acid oxidation in the liver [59,68]. Overall, our results provide multi-omics evidence that *L. rhamnosus* CU262 exerts comprehensive anti-obesity effects via concerted modulation of the gut microbiome and the resulting gut–liver metabolic signaling network, as summarized graphically in Figure 7.

## 4. Discussion

### 4.1. Key Findings and Theoretical Implications

This study demonstrated that *L. rhamnosus* CU262 significantly alleviates high-fat diet (HFD)-induced obesity and associated metabolic disturbances in mice. The effects showed a clear dose dependency, with the higher dose (10^9^ CFU) required to achieve significant metabolic improvements. CU262 supplementation effectively suppressed excessive body weight gain and adipose accumulation, improved the serum lipid profile (reducing triglycerides and LDL-cholesterol while elevating HDL-cholesterol), and attenuated systemic inflammation and oxidative stress markers [69]. Notably, anti-inflammatory cytokine levels (e.g., IL-10) were elevated and indices of liver injury were reduced in CU262-treated mice, indicating protection of hepatic function [70]. These findings corroborate evidence that probiotics can exert multi-faceted anti-obesity effects [9]. Theoretical implications of our results are grounded in the gut–liver axis paradigm: CU262’s benefits appear to stem from holistic modulation of gut microbial ecology and metabolic signaling to the host liver [71]. By integrating microbiome, short-chain fatty acid (SCFA) profiles, and liver transcriptomics, this work provides multi-omic evidence that targeting gut microbiota can induce systemic improvements in host energy and lipid metabolism [72]. This supports the concept that interventions in the gut (such as probiotic administration) can transmit signals to distal organs like the liver to improve metabolic homeostasis [71]. In summary, CU262 combats diet-induced obesity through coordinated improvements in gut microbial balance and host metabolic regulation, reinforcing the gut microbiome as a viable target for obesity intervention [73].

### 4.2. Mechanistic Insights into CU262’s Anti-Obesity Effects

CU262’s anti-obesity efficacy is mechanistically linked to its modulation of the gut microbiota and downstream metabolic pathways in the host liver [71]. HFD feeding in control mice resulted in gut dysbiosis, characterized by a significant loss of microbial diversity, an increased *Firmicutes*/*Bacteroidetes* ratio, and depletion of beneficial taxa (e.g., *Akkermansia* and butyrate-producing *Faecalibaculum*) [74]. These microbial alterations are consistent with prior observations that obesity is associated with an enrichment of energy-harvesting *Firmicutes* and a reduction in beneficial commensals [50]. CU262 supplementation reversed this dysbiosis: alpha-diversity of the gut microbiota was restored, the F/B ratio was lowered toward a lean-like state, and beneficial bacteria were markedly enriched [75]. In particular, the relative abundance of mucin-degrading *Akkermansia* was significantly elevated in CU262-treated mice, accompanied by increased fecal levels of total SCFAs (especially acetate and butyrate) [76]. This is a noteworthy finding because *Akkermansia* muciniphila has been widely reported as a key anti-obesity bacterium that strengthens gut barrier function and ameliorates metabolic inflammation [77]. Oral supplementation of *Akkermansia* in obese mice can reduce adiposity and improve metabolic health without affecting food intake, largely by improving intestinal barrier integrity and reducing endotoxemia [78]. The bloom of *Akkermansia* in the CU262 group likely contributed to the restoration of gut barrier function and reduction in systemic inflammation observed in our study [71].

Restoration of SCFA production is another critical mechanism [79]. HFD-fed control mice showed significantly lower cecal and fecal SCFA concentrations, a common consequence of microbiota dysbiosis under high-fat feeding [80]. CU262 intervention substantially increased SCFA levels [81], notably acetate and butyrate, consistent with the enrichment of SCFA-producing microbes [82]. This observation aligns with other probiotic studies; for instance, *L. rhamnosus* GG treatment of obese mice was shown to restore colonic propionate and butyrate levels diminished by HFD [82]. SCFAs are well-recognized signaling metabolites that can beneficially regulate host energy metabolism and immunity [83]. Acetate, for example, contributes to appetite regulation and lipid metabolism, helping maintain energy balance and body weight [79]. Propionate can act in the liver to inhibit cholesterol biosynthesis by suppressing HMG-CoA reductase activity and indeed has been shown to improve dyslipidemia and reduce atherosclerosis in obese mice [84]. Butyrate serves as the primary fuel for colonic epithelial cells and reinforces the intestinal barrier; it has demonstrated anti-inflammatory effects via activation of G-protein coupled receptors (FFAR2/3) and inhibition of histone deacetylases [85]. Thus, the SCFA profile improvement induced by CU262 likely contributed to enhanced gut barrier integrity, reduced systemic inflammation, and favorable shifts in host lipid metabolism (including better cholesterol homeostasis) [45]. In our correlation analyses, the levels of fecal acetate and butyrate were inversely correlated with serum lipids and liver weight, supporting a causal role for SCFAs in mediating CU262’s metabolic benefits [86].

At the molecular level, CU262 profoundly affected host hepatic gene expression [87]. HFD feeding drove the hepatic transcriptome toward a pro-obesity state, with significant upregulation of genes involved in cholesterol biosynthesis (the mevalonate pathway) and endoplasmic reticulum (ER) stress [57]. Key enzymes of the cholesterol synthesis pathway—*Mvk*, *Mvd*, *Fdps*, *Nsdhl*, and *Dhcr7*—were markedly upregulated in HFD control mice, indicating activation of the mevalonate pathway and excessive cholesterol production [88]. Concordantly, *Pcsk9* was upregulated by HFD, which would lead to increased *PCSK9*-mediated degradation of hepatic LDL receptors, impairing LDL-cholesterol clearance [89]. These molecular disturbances are characteristic of diet-induced hypercholesterolemia and steatosis: HFD has been reported to elevate *PCSK9* expression, contributing to reduced hepatic LDL uptake and hyperlipidemia [90]. Strikingly, CU262 supplementation reversed these pathological gene expression changes [91,92]. In CU262-treated mice, the transcripts for *Mvk*, *Mvd*, *Fdps*, *Nsdhl* (and the related *Dhcr7*) were all significantly downregulated relative to HFD controls, suggesting that de novo cholesterol synthesis in the liver was attenuated [93]. *Pcsk9* expression was also markedly reduced by CU262, a change predicted to enhance hepatic LDLR recycling and clearance of circulating LDL-C [94]. This mechanism is analogous to the action of *PCSK9*-inhibitor drugs, which are known to boost hepatic LDL uptake and thereby reduce blood cholesterol [95]. The downregulation of *PCSK9* by a probiotic is a novel and noteworthy finding, as it links gut microbial intervention to host cholesterol homeostasis through a key regulatory node [94]. Additionally, CU262 normalized the expression of multiple genes associated with ER stress and the unfolded protein response (e.g., *Hspa1b*, *Hyou1*, *Manf*) [96]. Since unresolved ER stress in obesity drives hepatic lipogenesis and inflammatory signaling, the alleviation of ER stress by CU262 would contribute to reduced lipid synthesis and improved insulin sensitivity in the liver [97]. Taken together, these transcriptomic changes illustrate a multi-pronged mechanism: CU262 curtails pathological cholesterol overproduction and may improve lipid clearance (via the *PCSK9*-LDLR axis) while also easing ER stress and its metabolic consequences [98]. Such “gene–enzyme–pathway” level modulations by CU262 underscore the deep impact of gut microbiota on host metabolic pathways [99].

Pathway enrichment analyses reinforced these insights [57]. Gene set enrichment analysis (GSEA) revealed that in HFD vs. control livers, cholesterol/steroid biosynthesis pathways were highly upregulated (positively enriched), reflecting the obesogenic stimulus [88]. In contrast, in CU262-treated vs. untreated HFD livers, the cholesterol biosynthesis pathways showed significant negative enrichment, indicating that CU262 actively suppressed these pathways [100]. Concurrently, CU262 administration led to enrichment of pathways related to oxidative phosphorylation, fatty acid β-oxidation, glutathione metabolism, and drug metabolism, all of which are associated with enhanced mitochondrial function and detoxification/antioxidant capacity [101]. The upregulation of these protective pathways is consistent with the observed reductions in oxidative stress markers and improved liver function in CU262-treated mice [102]. Notably, the glutathione metabolism pathway enrichment suggests an improved hepatic antioxidant defense, which would help counteract HFD-induced oxidative damage [101]. Overall, these multi-omics results support a comprehensive mechanistic model: CU262 remodels the gut microbiota and increases SCFA release, which in turn modulates host signaling (e.g., via *FFAR2/3* and other mechanisms) to downregulate hepatic cholesterol synthesis and inflammation while upregulating fatty acid oxidation and antioxidant defenses [103]. This orchestrated modulation across the gut–liver axis ultimately restores metabolic homeostasis, illustrating how a probiotic can exert systemic anti-obesity effects through interconnected biological networks [71].

Our findings on CU262 align with and add to a growing literature on probiotic interventions in obesity. Recent studies have shown that both single-strain and multi-strain probiotic formulations can confer anti-obesity benefits in HFD-induced models [104]. For example, a four-strain mixture of lactobacilli (including *L. plantarum, L. paracasei*, *L. rhamnosus* GG, and *L. sakei,* termed *L-PPRS*) effectively prevented HFD-induced obesity in mice [70]. Similarly to CU262, the L-PPRS treatment significantly inhibited weight gain, improved glucose tolerance and insulin sensitivity, lowered serum lipid levels, and reduced fat deposition in both adipose tissue and liver [70]. The multi-strain probiotic also alleviated HFD-induced oxidative stress in the liver and exerted anti-inflammatory effects, paralleling our observations with CU262 [105]. Notably, *L-PPRS* restored gut microbiota homeostasis by decreasing the F/B ratio and enriching beneficial microbes, which is highly consistent with the microbiota modulation we observed [70]. The convergence of outcomes between CU262 and *L-PPRS* underscores a common probiotic action mode: rebalancing the gut ecosystem and metabolic signaling to counteract diet-induced metabolic derangements [106].

Comparative evidence is also available for *L. rhamnosus* strains in mono- or duo-therapy [36]. Recent research demonstrated that *L. rhamnosus* LS-8, either alone or combined with another commensal (*L. crustorum*), ameliorated obesity and metabolic disturbances in mice fed a high-fat/high-fructose diet [107]. The treated mice showed improved insulin sensitivity and decreased systemic inflammation, and importantly, their gut microbiota shifted favorably: pathogenic genera like *Bacteroides* and *Desulfovibrio* declined, while beneficial *Lactobacillus* and *Bifidobacterium* populations increased, along with elevated fecal SCFA levels [108]. This phenotype closely mirrors the effects of CU262 on restoring a healthier microbial and metabolic profile [70]. Another study found that *L. rhamnosus* 4B15 combined with a prebiotic (hydrolyzed lactose skim milk) reduced weight gain and adiposity in HFD-fed rats, which was attributed to downregulation of lipogenic genes and upregulation of fatty acid oxidation pathways [109]. Likewise, *L. rhamnosus* GG has been shown by multiple groups to improve metabolic outcomes in obese models—for instance, improving insulin sensitivity and increasing adiponectin levels while reducing adipose inflammation [110]. Our results extend these prior studies by providing a more detailed mechanistic picture (through multi-omics) and by identifying a novel strain (CU262) with robust anti-obesity efficacy.

It is also worth noting that our integrative approach (combining microbiota, metabolite, and transcriptome analysis) is in line with emerging trends in probiotic research. Recent reviews highlight that advanced omics techniques are uncovering how probiotics modulate host pathways across the gut–liver axis [111]. For example, a multi-omics analysis of a *Lactobacillus* plantarum intervention in diabetic mice revealed that its anti-metabolic syndrome effects were mediated through gut microbiota shifts and hepatic transcriptional changes, much like the CU262 findings [87]. Thus, our study not only corroborates the anti-obesity impacts of certain probiotic strains reported in the literature but also provides a comprehensive mechanistic framework that complements and deepens insights from recent studies [112]. By comparing CU262’s effects with those reported for other probiotics, we can conclude that modulating the gut microbiome (especially increasing beneficial microbes and their metabolites) and targeting host metabolic pathways (lipid metabolism, inflammation, etc.) are common, possibly synergistic strategies among probiotics to combat obesity [70]. CU262 stands out by significantly influencing the cholesterol metabolic axis (mevalonate pathway and *PCSK9*)—a feature that is not widely documented for many probiotics—suggesting that strains may have strain-specific “signatures” in their mechanism, an important consideration for probiotic selection in metabolic disease intervention [101].

### 4.3. Theoretical Contributions and Potential Functional Food Applications

Beyond the empirical findings, this work makes several theoretical contributions to the understanding of probiotics as metabolic modulators [113]. First, it reinforces the concept of the gut–liver axis as a critical pathway in obesity pathogenesis and therapy [71]. While previous studies have acknowledged the role of gut microbes in obesity, our multi-omics evidence explicitly maps how a probiotic intervention in the gut can reverberate through microbial metabolites to influence gene expression in the liver. This cascade ultimately culminates in systemic metabolic improvements [114]. This integrated perspective advances the theoretical framework that metabolic diseases like obesity are not solely driven by caloric excess but also by dysregulated host–microbiome interactions [115]. By elucidating CU262’s mechanisms, we contribute to a more nuanced model wherein specific microbial changes (e.g., restoring *Akkermansia* and SCFA producers) are linked to specific host responses (e.g., reduced cholesterol synthesis and inflammation) [73]. Such knowledge deepens scientific understanding of strain-specific probiotic actions—an important theoretical point since not all probiotics are equal, and the efficacy often depends on unique traits of the strain [116]. Our study highlights that CU262 can influence host cholesterol metabolism and ER stress, suggesting theoretical avenues whereby probiotics might be leveraged to target cardiovascular risk factors (like hypercholesterolemia) in addition to obesity itself [117].

In terms of practical implications, these findings open up possibilities for developing CU262 as a functional food ingredient or nutraceutical for obesity intervention [112]. *L. rhamnosus* is generally regarded as safe (GRAS) and has a long history of use in fermented foods and supplements, which bodes well for translating CU262 into a functional product [118]. The strain CU262, originally isolated from infant gut flora, could be formulated into probiotic yogurts, drinks, or capsules aimed at weight management and metabolic health improvement [16]. The multi-dimensional benefits observed—including weight reduction, lipid-lowering, anti-inflammatory, and liver-protective effects—indicate that CU262 could serve as a valuable component of a multifaceted strategy to combat metabolic syndrome [119]. For instance, CU262 might be incorporated into a functional dairy product designed for individuals with obesity or metabolic-associated fatty liver disease (MAFLD) as an adjunct to dietary and lifestyle modifications [39]. Its capacity to increase SCFAs is particularly advantageous, as these metabolites not only have local gut benefits but may also induce satiety hormones (such as PYY/GLP-1) and improve insulin sensitivity, thereby aiding in weight control [120]. Additionally, the apparent effect on *PCSK9* and cholesterol metabolism raises the effect on *PCSK9* and hints at a potential cholesterol-lowering mechanism, but direct evidence is lacking, and further research is required to evaluate any clinical implications [95].

We note that the modulation of *Pcsk9* by CU262 suggests a potential lipid-lowering capacity; however, direct evidence is currently lacking, and its clinical applicability requires further investigation. Another practical aspect is the potential development of postbiotic or para-probiotic products based on CU262 [121]. Recent research suggests that even inactivated probiotic cells or cell-free supernatants can retain beneficial effects [110]. If CU262’s key effector components (for example, secreted metabolites or cell wall fragments) can be identified, one could design functional foods that deliver these components without requiring live bacteria, which simplifies storage and safety considerations [122]. This approach could widen the applicability of CU262 in various food matrices (including heat-treated products) while still conferring metabolic benefits [123]. Overall, our study provides a strong proof-of-concept that CU262 is an efficacious candidate for functional food applications in the context of obesity and metabolic syndrome [112]. The evidence generated can be used to support regulatory approval and consumer acceptance of products containing CU262 or its derivatives [118]. In contributing both mechanistic insight and practical leads, this work bridges the gap between fundamental probiotic research and real-world nutritional strategies for obesity management [124].

### 4.4. Limitations and Future Directions

While the results of this study are promising, several limitations should be acknowledged, and they point to directions for future research [125].

First, the study was conducted in a controlled HFD-induced obesity mouse model with a relatively small sample size (*n* = 6 per group) [126]. However, the translatability of findings to humans remains to be established [125]. Translating the effective mouse dose of CU262 (10^9^ CFU per day) to humans suggests a human-equivalent dose on the order of 10^11^ CFU or higher per day for an adult, assuming linear scaling by body mass. Such doses are within feasible ranges, as human probiotic products often contain ~10^10^ CFU or more per serving. Future studies could include larger cohorts and different animal models (such as genetic models of obesity or dyslipidemia) to confirm the reproducibility of CU262’s effects, and human trials are needed to validate both safety and effectiveness [126].

In addition, it should be noted that this study was conducted only in male mice. Metabolic and immunological responses to high-fat diets and probiotics can differ between sexes due to hormonal and physiological differences. Since we have not yet tested CU262 in female mice, the applicability of our findings to females remains to be confirmed. To ensure broader applicability, we plan to examine the effects of CU262 in female mouse models in future experiments to determine whether similar anti-obesity and hepatoprotective benefits occur. Such studies will be important to ensure that the probiotic’s efficacy is not sex-specific [127].

A further limitation is that food intake was not recorded for the different groups. Thus, we cannot completely rule out the possibility that CU262-treated mice consumed fewer calories, which might have contributed to their reduced weight gain and improved metabolic profiles. Although we did not observe obvious differences in feeding behavior, the lack of quantitative data means that a potential, subtle reduction in food consumption remains a confounding factor. Future studies should monitor food consumption to distinguish direct metabolic effects of the probiotic from any intake-mediated effects.

Moreover, our study lasted 12 weeks. It is currently unclear how persistently CU262 colonizes the gut after cessation of dosing and whether the gut–liver axis reprogramming remains stable without continued probiotic supplementation. Because our protocol involved sacrificing the mice at week 12 with no post-treatment follow-up period, we could not assess the longevity of CU262’s colonization or the durability of its effects. This is an important question for future studies. It may be that continuous intake of CU262 is required to maintain the beneficial metabolic effects observed, as probiotic colonization is often transient [128]. Long-term safety is another consideration; although *L. rhamnosus* species are generally safe, chronic use should be evaluated.

Second, although our multi-omics approach uncovered strong correlations between gut microbiota changes, SCFA levels, and host gene expression, the causal relationships warrant further investigation [129]. For example, future studies could use germ-free or antibiotic-treated mouse models to confirm that the gut microbiota is necessary for the anti-obesity effects of CU262 [115]. Additionally, blocking SCFA receptors (FFAR2/3) or using knockout models could directly test whether SCFA signaling is a causal mediator of the metabolic improvements [130]. Such mechanistic studies would solidify the link between CU262-induced SCFA production and host metabolic regulation [131].

Furthermore, although CU262 treatment was associated with reduced systemic inflammation (as evidenced by lower IL-6/TNF-α and higher IL-10 levels), we did not directly assess intestinal barrier function in this study. Key markers of gut permeability (e.g., tight junction proteins like zonulin/occludin) or endotoxin translocation (circulating lipopolysaccharide) were not measured, which is a limitation of our work. Therefore, while improved barrier integrity is a plausible mechanism underlying the probiotic’s effects (supported by literature on probiotics strengthening the gut barrier), our results do not explicitly demonstrate such an effect. Future investigations should include measurements of intestinal permeability and endotoxemia to determine whether the CU262 isolate directly fortifies the gut barrier and prevents endotoxin leakage into the circulation.

Additionally, our analysis focused on gene expression and did not measure protein levels or enzyme activities for key pathways, e.g., it remains to be determined whether the observed downregulation of *Pcsk9* mRNA in the liver translates into reduced circulating *PCSK9* protein. We did not measure serum *PCSK9* in this study and therefore cannot confirm a protein-level change. [132]. Future studies should include an assay (e.g., ELISA) for *PCSK9* protein to verify that CU262’s effect on gene expression leads to functionally significant changes in cholesterol metabolism [133]. Also, we will further validate representative DEGs by RT-qPCR in follow-up studies. It would also be insightful to perform targeted metabolomic analyses of the liver and serum to identify other microbial metabolites (beyond SCFAs) altered by CU262—for instance, bile acid profiles or tryptophan metabolites, which also participate in gut–liver signaling [134].

It is important to note that the lower serum LDL-C levels observed in CU262-treated mice could arise from mechanisms beyond *PCSK9* downregulation. For example, probiotics (including lactic acid bacteria) are known to influence bile acid metabolism and fecal cholesterol excretion, which can reduce circulating cholesterol independently of the PCSK9-LDLR axis. Additionally, the overall reduction in adiposity and hepatic lipogenesis (evidenced by downregulation of mevalonate pathway genes) in the treated mice likely contributed to improved lipid profiles. Therefore, while the decrease in hepatic *Pcsk9* expression suggests enhanced LDL receptor availability, we acknowledge that this is an indirect inference. Without direct protein-level data (e.g., PCSK9 protein or LDLR levels) or functional assays, the therapeutic implications of *PCSK9* modulation remain speculative. We have thus avoided overstating this point and instead emphasize that multiple factors, possibly including SCFA-mediated inhibition of cholesterol synthesis and probiotic effects on intestinal lipid absorption, could collectively explain the LDL-C reduction.

Finally, exploring CU262’s genome and functional genomics could provide insight into what makes this strain particularly effective [17]. Comparative genomics with other *L. rhamnosus* strains might reveal unique genes (perhaps related to bile salt hydrolase activity, SCFA production, or immunomodulation) that confer the observed metabolic benefits [17]. Such information could guide bioengineering approaches to further enhance probiotic strains. In conclusion, our study provides a robust foundation for the development of CU262 as an anti-obesity probiotic.

In terms of future directions, one exciting avenue is to test CU262 in clinical trials or human settings [112]. An initial trial could involve overweight/obese individuals receiving CU262 as a dietary supplement to see if it yields improvements in weight control, lipid profiles, inflammatory markers, and perhaps liver enzymes (for MAFLD indicators) [38]. Even a short-term human study could examine if CU262 consumption modulates the gut microbiome similarly to in mice (e.g., increases in *Akkermansia* and SCFA producers) and is well-tolerated [73]. Another direction is exploring synergistic interventions: CU262 might be combined with dietary fiber or polyphenols that also target the gut microbiota, potentially amplifying the beneficial SCFA production and metabolic effects [38]. Additionally, given the indication that even heat-killed CU262 or its metabolites might retain efficacy (as suggested by analogous findings with postbiotics), research could focus on isolating bioactive compounds from CU262 [135]. Identifying specific molecular effectors (such as exopolysaccharides, peptides, or enzymes) could lead to novel therapeutic agents or more stable functional food ingredients [136].

In conclusion, our study provides a robust foundation for the development of CU262 as an anti-obesity probiotic, but future research should address current limitations through in-depth mechanistic investigations and long-term translational studies, while well-designed clinical trials are essential to validate its efficacy in patients and advance CU262 as a novel gut-liver axis-targeted intervention against obesity and MAFLD [137]. These efforts will help in fully validating CU262’s therapeutic potential and in paving the way from experimental findings to tangible health interventions for obesity and related metabolic disorders [38].

## 5. Conclusions

In summary, *Lacticaseibacillus rhamnosus* CU262 exhibited robust anti-obesity and liver-protective effects in HFD-fed mice through multi-faceted mechanisms across the gut–liver axis. At the hepatic level, CU262 intervention broadly suppressed cholesterol biosynthesis and alleviated endoplasmic reticulum stress, indicating improved hepatic metabolic homeostasis (including enhanced cholesterol clearance) and mitigation of key factors driving fatty liver progression. Concurrently, CU262 reshaped the gut microecology by restoring a healthier microbiota composition. It enriched beneficial short-chain fatty acid (SCFA)-producing taxa such as *Akkermansia* (often depleted in obesity and linked to metabolic health) and elevated fecal SCFA levels (notably acetate and butyrate). These microbial and metabolite shifts likely fortify the intestinal barrier and stimulate hepatic fatty acid oxidation and energy expenditure, thereby lowering liver fat accumulation and inflammation. Together, these hepatic and gut-level changes illustrate how CU262 modulated the gut–liver axis to counteract HFD-induced metabolic derangements.

Collectively, our multi-omics findings underscore the translational potential of *L. rhamnosus* CU262 as a functional probiotic for obesity-related metabolic disorders through gut–liver axis modulation. By simultaneously improving gut microbiota balance and reprogramming host hepatic pathways, CU262 addressed multiple pathological aspects of diet-induced metabolic syndrome and metabolic-associated fatty liver disease (MAFLD). This multi-targeted approach aligns with emerging evidence that probiotics can safely ameliorate metabolic dysfunction—improving lipid profiles, reducing hepatic steatosis, and attenuating systemic inflammation—in both preclinical models and human studies. As a naturally safe microbial therapy with minimal side effects, CU262 could serve as an attractive dietary supplement or adjunct treatment for managing metabolic syndrome and MAFLD. Although clinical validation is needed to confirm these benefits in patients, the multi-omics insights provided here offer a strong scientific foundation for developing CU262 into a novel probiotic intervention that harnesses microecological regulation of the gut–liver axis to combat metabolic dysfunction.

## Data Availability

The original contributions presented in this study are included in the article/Supplementary Materials. Further inquiries can be directed to the corresponding authors.

## Supplementary Materials

The following supporting information can be downloaded at: https://www.mdpi.com/article/10.3390/foods15020332/s1, Table S1: Major reagents; Table S2: Statistics of Figure S5; Table S3: Statistics of Figure S6.
