# Supplementary material for: Lacticaseibacillus rhamnosus CU262 Attenuates High-Fat Diet–Induced Obesity via Gut–Liver Axis Reprogramming
Source: Foods. 2026 Jan 16;15(2):332. doi: 10.3390/foods15020332 (PMC12841443; doi:10.3390/foods15020332)
Supplement: Supplementary file 1 [file foods-15-00332-s001.zip › Table S1.pdf]

Table S1. Major reagents

Name	Producer
Phosphoric acid	Shanghai Sinopharm Chemical Reagent Co., Ltd.
4-Methylvaleric acid	Shanghai Sinopharm Chemical Reagent Co., Ltd.
Diethyl ether	Shanghai Sinopharm Chemical Reagent Co., Ltd.
Anhydrous ethanol	Tianjin Fuyu Fine Chemical Co., Ltd.
Total Cholesterol (TC) Assay Kit	Nanjing Jiancheng Biological Engineering Research Institute
Triglyceride (TG) Assay Kit	Nanjing Jiancheng Biological Engineering Research Institute
Low-Density Lipoprotein Cholesterol (LDL-C) Assay Kit	Nanjing Jiancheng Biological Engineering Research Institute
High-Density Lipoprotein Cholesterol (HDL-C) Assay Kit	Nanjing Jiancheng Biological Engineering Research Institute
Alanine Aminotransferase (ALT) Assay Kit	Nanjing Jiancheng Biological Engineering Research Institute
Aspartate Aminotransferase (AST) Assay Kit	Nanjing Jiancheng Biological Engineering Research Institute
Total Bile Acid (TBA) Assay Kit	Nanjing Jiancheng Biological Engineering Research Institute
Catalase (CAT) Assay Kit	Nanjing Jiancheng Biological Engineering Research Institute
Total Superoxide Dismutase (SOD) Assay Kit	Nanjing Jiancheng Biological Engineering Research Institute
Malondialdehyde (MDA) Assay Kit	Nanjing Jiancheng Biological Engineering Research Institute
Interleukin-6 (IL-6) ELISA Kit	Shenzhen NeoBioscience Technology Co., Ltd.
Interleukin-10 (IL-10) ELISA Kit	Shenzhen NeoBioscience Technology Co., Ltd.
Tumor Necrosis Factor-α (TNF-α) ELISA Kit	Shenzhen NeoBioscience Technology Co., Ltd.
Gram Staining Kit	Guangdong Huankai Microbial Sci. & Tech. Co., Ltd.
Free Fatty Acid (FFA) Content Assay Kit	Shanghai ZCIBIO Technology Co., Ltd.
